# Fetal posterior fossa malformations: review of the current knowledge

**DOI:** 10.1590/0100-3984.2018.0141

**Published:** 2019

**Authors:** Hérbene Jose Figuinha Milani, Enoch Quindere de Sá Barreto, Renato Luis da Silveira Ximenes, Carlos Alberto Raimundo Baldo, Edward Araujo Júnior, Antonio Fernandes Moron

**Affiliations:** 1 Department of Obstetrics, Escola Paulista de Medicina da Universidade Federal de São Paulo (EPM-Unifesp), São Paulo, SP, Brazil.; 2 Centrus - Diagnostic Imaging, Campinas, SP, Brazil.

**Keywords:** Prenatal diagnosis/methods, Ultrasonography/methods, Cranial fossa, posterior/abnormalities, Cerebellum/embryology

## Abstract

Ultrasound diagnosis of posterior fossa malformations in the prenatal period is a challenge, having major implications for the counseling and follow-up of pregnant women. The purpose of this study was to review aspects of the ultrasound evaluation of the fetal posterior fossa, as well as to describe the most relevant ultrasound findings of the main posterior fossa malformations that can affect the fetus in the prenatal period.

## INTRODUCTION

Ultrasound diagnosis of posterior fossa malformations in the prenatal period is a challenge, having major implications for the counseling and follow-up of pregnant women. These malformations encompass a wide spectrum of entities, ranging from normal variants to severe anomalies, often with similar aspects on fetal ultrasound; a variety of terms are used in order to describe these anomalies, without a uniform approach to their description; and the method of evaluating the posterior fossa structures during gestation, which is routinely performed in the axial planes of the fetal skull by ultrasound, is inappropriate^(1-3)^.

The objective of this study was to review aspects of ultrasound evaluation of the fetal posterior fossa, with an emphasis on the neurosonographic aspects, as well as to describe the most relevant prenatal ultrasound findings of the main posterior fossa malformations that can affect the fetus.

## ULTRASOUND EVALUATION OF THE FETAL POSTERIOR FOSSA

During gestation, the fetal brain, including the structures of the posterior fossa, undergoes more changes than does any other organ. Therefore, before starting to evaluate the posterior fossa during the prenatal period, the professionals involved should be familiar with the developmental (anatomical and embryological) aspects of those structures, with the objective of diagnosing deviations in their formation, avoiding any confusion between normal aspects of development and possible malformations.

The posterior fossa is composed primarily of the following structures: the cerebellum (comprising the cerebellar hemispheres and vermis); the cerebral peduncles; the fourth ventricle; the brainstem (pons and bulb); the cisterna magna; and the tentorium. Embryologically, the structures of the posterior fossa are derived from the hindbrain (rhombencephalon), which differentiates into the metencephalon and myelencephalon. The cerebellum, pons, and upper portion of the fourth ventricle arise from the metencephalon, whereas the bulb and lower portion of the fourth ventricle arise from the myelencephalon. In the sixth week of gestation, the pontine flexure arises, with formation of the anterior and posterior membranous areas. The anterior membranous area gives rise to the cerebellar vermis, and the posterior membranous area gives rise to the cisterna magna and a recess known as Blake’s pouch. The latter disappears between weeks 16 and 18 with the fenestration of the fourth ventricle (formation of the foramina of Luschka and Magendie) and its closure^(4,5)^.

For ultrasound evaluation of the fetal posterior fossa, it is important to keep in mind that Blake’s pouch is a normal embryological structure of the onset of fetal development that often disappears at approximately week 18 of gestation, with closure of the fourth ventricle (i.e., when there is no longer any communication between the fourth ventricle and the cisterna magna). However, communication between the fourth ventricle and the cisterna magna can still be identified by ultrasound up to week 20 of gestation. Cerebellar foliations and vermis fissures are formed as of the fifth month of gestation, the primary fissure of the vermis being identified in 100% of cases only after week 24 of gestation^(6)^. Therefore, the early diagnosis of posterior fossa malformations is often very difficult, due to the characteristics of the formation of these structures^(7)^.

Ultrasound is the method of choice for the screening and diagnosis of malformations of the fetal central nervous system, including those of the posterior fossa. Since 2007, the International Society of Ultrasound in Obstetrics and Gynecology (ISUOG) has recommended perform a basic evaluation (screening) as well as neurosonographic evaluation of these structures^(8)^.

**Figure 1:**
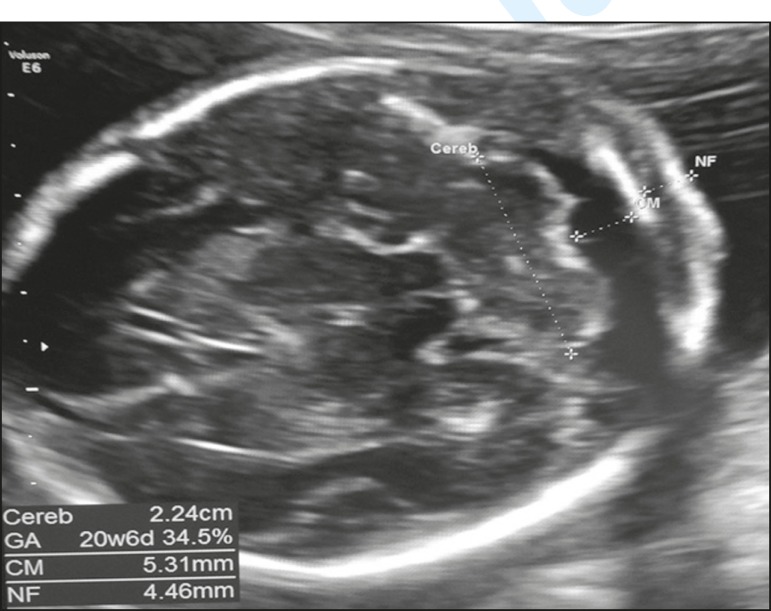
Axial ultrasound of the transcerebellar plane, evaluating the cerebellar hemispheres (shape and contours); the vermis (more highly echogenic structure between the two cerebellar hemispheres); the biometry of the cerebellum (transcerebellar diameter); the shape and transverse diameter of the cisterna magna; and the size of the fourth ventricle.

In routine screening, ultrasound images of the posterior fossa malformations are obtained in an axial view of the transcerebellar plane, with the transducer positioned over the abdomen of the pregnant woman. The following aspects are evaluated in that plane (Figure 1): the cerebellar hemispheres (shape and contours); vermis (more highly echogenic structure between the two cerebellar hemispheres); the cerebellum (transcerebellar diameter, by biometry); and the shape and transverse measurement of the cisterna magna. However, multiplanar evaluation of these structures (including images obtained in the sagittal and coronal planes) is necessary for the differential diagnosis of posterior fossa malformations, which, according to the ISUOG guidelines, should be part of the neurosonographic evaluation. A multiplanar approach is the basis of the neurosonographic examination of the fetal brain, which is performed by aligning the transducer with the sutures and fontanelles of the fetal head. When the fetus is in vertex presentation, a transvaginal approach can be used. Vaginal probes have the advantage of operating at a higher frequency than do abdominal probes and therefore allow greater definition of anatomical details^(8)^.

**Figure 2:**
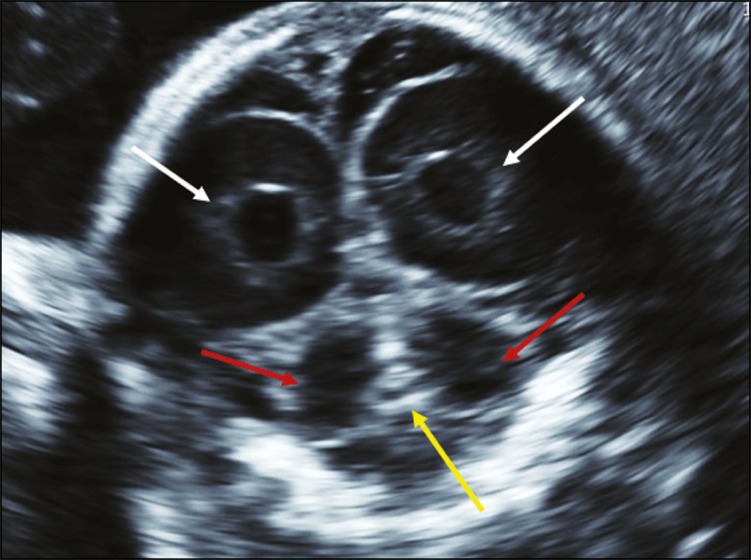
Coronal ultrasound of the transcerebellar plane, evaluating the cerebellum (shape, contours, and foliations of the cerebellar hemispheres); the vermis (more highly echogenic structure located between the two cerebellar hemispheres, yellow arrow). Coronal imaging is very important for differentiating between the cerebellar hemispheres and the vermis.

In the coronal view of the transcerebellar plane (Figure 2), the following structures are evaluated: cerebellum (shape, contours, and foliations of the cerebellar hemispheres); vermis (more highly echogenic structure located between the two cerebellar hemispheres). This plane is very important for differentiation between the cerebellar hemispheres and vermis, which facilitates the diagnosis of vermian agenesis.

**Figure 3:**
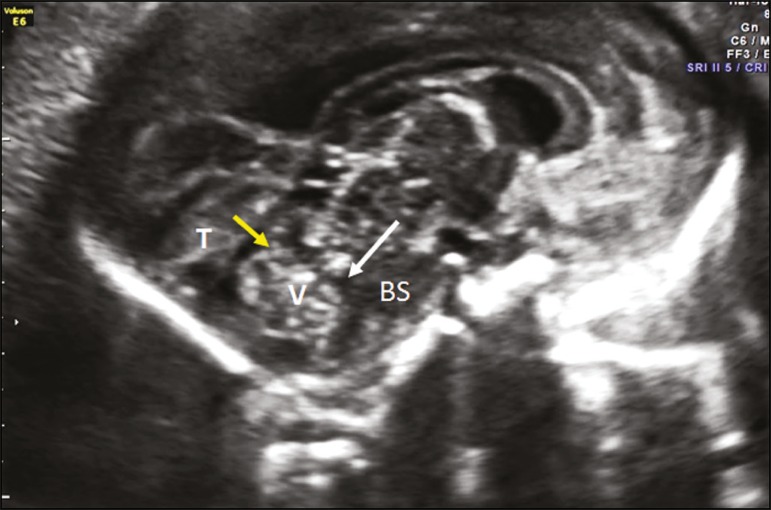
Ultrasound in the median sagittal plane, the best plane in which to assess the fetal posterior fossa (mainly for the differential diagnosis of cystic malformations). It is possible to identify the following: the brainstem (BS); the cerebellar vermis (V), with assessment of its morphology and biometry (craniocaudal and anteroposterior diameter); the primary fissure (yellow arrow); the fourth ventricle; the fastigium (white arrow); and the tentorium (T).

**Figure 4:**
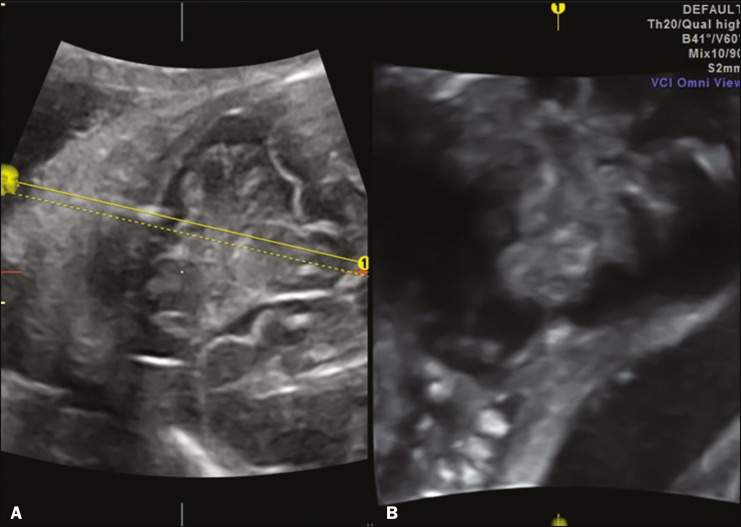
3D ultrasonography of the posterior fossa at 28 weeks of gestation, which allows multiplanar evaluation (OmniView). **A:** Axial acquisition in the transcerebellar plane. **B:** Reconstruction in the sagittal plane (OmniView) of the posterior fossa.

Undoubtedly, the most important plane for evaluating the fetal posterior fossa by ultrasound (mainly for the differential diagnosis of cystic malformations of the posterior fossa) is the median sagittal plane. In this plane (Figure 3), it is possible to identify the brainstem (pons and bulb, with measurement of the diameter of the pons); to assess the morphology of the cerebellar vermis by biometry (craniocaudal and anteroposterior diameters); to evaluate the fourth ventricle and its fastigium; to identify the primary fissure, which divides the vermis into its superior and inferior portions, being an important marker for changes in its development (the ratio between the superior and inferior portions is usually 1:2), and should be identified in 100% of cases after week 24 of gestation; to evaluate the cisterna magna (shape and diameter); and to determine the position of the tentorium, which is an important marker for the differential diagnosis of cystic malformations of the posterior fossa. It is also possible to quantify the upward rotation of the cerebellar vermis and tentorium by measuring two angles^(1,9)^: that between the pons and the vermis; and that between the pons and the tentorium. Three-dimensional (3D) ultrasound can also be a useful tool in this evaluation because it allows multiplanar evaluation (through the use of 3D applications such as multiplanar, volume contrast imaging, and OmniView), as well as allowing storage of the volumes and their offline analysis at specialized centers (Figure 4).

## ULTRASOUND DIAGNOSIS OF POSTERIOR FOSSA MALFORMATIONS IN THE PRENATAL PERIOD

As previously mentioned, three fundamental factors hinder the ultrasound diagnosis of posterior fossa malformations: such malformations encompass a broad spectrum of different entities, ranging from normal variants to severe anomalies, which can present with similar findings on fetal ultrasound; the terminology employed in describing these anomalies varies; and the method of evaluating the posterior fossa structures during gestation, which is routinely performed in the axial planes of the fetal skull by ultrasound, is inappropriate. Therefore, to diagnose these malformations, it is necessary to be familiar with the main anomalies that affect the posterior fossa and their sonographic manifestations; to standardize the classification of posterior fossa malformations, the most widely accepted classification being that devised by Malinger et al.^(2)^, which includes cystic and non-cystic malformations, avoiding the term Dandy-Walker variant (which would include different entities with different prognoses); and to perform multiplanar evaluation of the posterior fossa structures (including the axial, sagittal, and coronal planes) as previously described.

**Figure 5:**
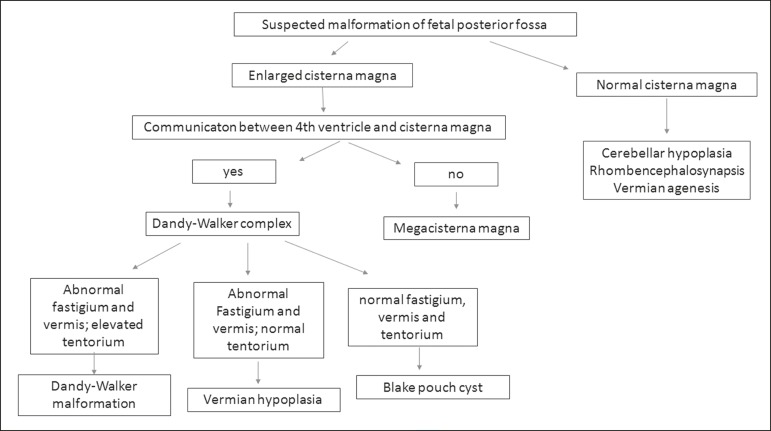
Flowchart for the differential diagnosis of posterior fossa anomalies.

In accordance with the Malinger et al.^(2)^ classification, we describe the main malformations of the posterior fossa that can affect the fetus, together with their ultrasound presentations. To aid in the differential diagnosis of posterior fossa anomalies, we have created a flowchart (Figure 5). The Chiari II malformation (an anomaly related to spina bifida) will not be described in this article.

Posterior fossa malformations can be initially divided into two groups: those in which the cisterna magna is enlarged and those in which it is normal. If the cisterna magna is enlarged, communication between the fourth ventricle and the cisterna magna should be investigated; if such communication is identified, Dandy-Walker complex malformations should be suspected; if not, mega-cisterna magna should be suspected. The differential diagnosis will therefore be based on evaluation in the median sagittal plane: if the fastigium and vermis are abnormal and the tentorium is elevated, Dandy-Walker malformation should be suspected; if the fastigium and vermis are abnormal and the tentorium is in the normal position, vermian hypoplasia should be suspected; if the fastigium, vermis, and tentorium are all normal, Blake’s pouch cyst should be suspected.

### Dandy-Walker complex

**Figure 6:**
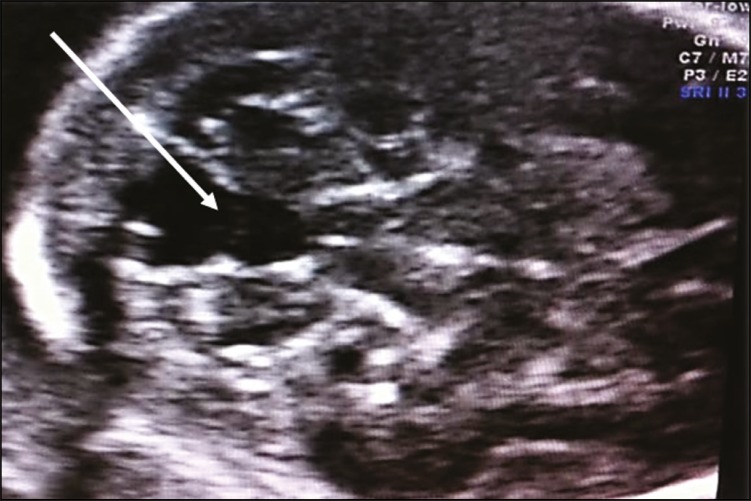
Axial ultrasound of the transcerebellar plane, showing communication between the fourth ventricle and the cisterna magna (arrow).

The three entities described below correspond to the Dandy-Walker complex. In the axial plane-only evaluations, they present similar ultrasound findings (communication between the fourth ventricle and the cisterna magna, as depicted in Figure 6), evaluation in the median sagittal plane therefore being fundamental for the differential diagnosis.

**Figure 7:**
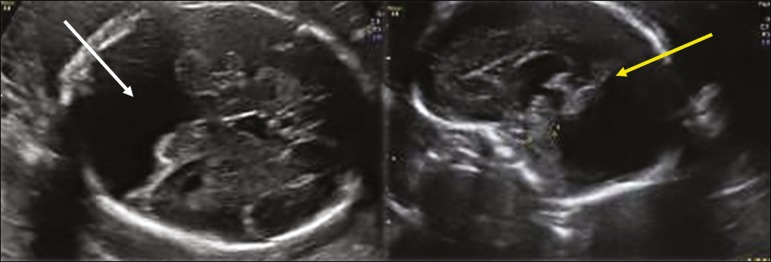
Axial ultrasound of a Dandy-Walker malformation, showing communication between the fourth ventricle and the cisterna magna (white arrow). Sagittal ultrasound showing an abnormal fastigium, vermian agenesis/hypoplasia with upward rotation, and elevation of the tentorium (yellow arrow).

***Dandy-Walker malformation* -** A Dandy-Walker malformation is defined as cystic dilation of the fourth ventricle associated with agenesis/hypoplasia of the cerebellar vermis and elevation of the tentorium. Axial ultrasound shows communication between the fourth ventricle and cisterna magna, whereas sagittal images show an abnormal fastigium, vermian agenesis/hypoplasia with upward rotation, and elevation of the tentorium (Figure 7). It is often accompanied by other malformations of the central nervous system, such as alterations in the corpus callosum and interhemispheric cysts.

**Figure 8:**
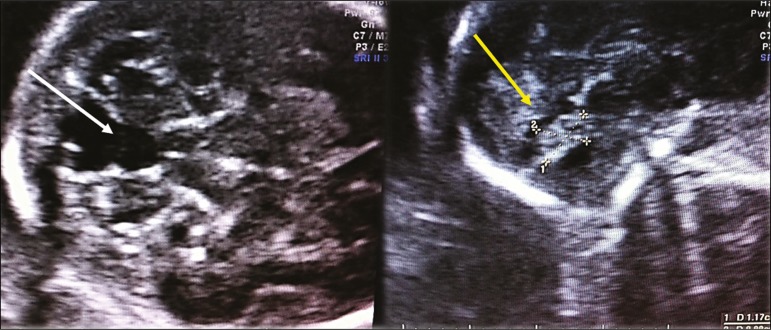
Axial ultrasound of a fetus with vermian hypoplasia, showing communication between the fourth ventricle and the cisterna magna (white arrow). Sagittal ultrasound showing a small vermis, an abnormal fastigium, and the tentorium in a normal position (yellow arrow).

***Vermian hypoplasia* -** In vermian hypoplasia, the vermis is normal in form but small in size. Axial ultrasound shows communication between the fourth ventricle and the cisterna magna. Sagittal ultrasound shows a small vermis (craniocaudal and anteroposterior diameters small for gestational age), an abnormal fastigium, and normal positioning of the tentorium (Figure 8).

***Blake’s pouch cyst* -** A Blake’s pouch cyst presents as an apparent communication between the cisterna magna and fourth ventricle, although the vermis and fastigium are normal. Ultrasound shows communication between the fourth ventricle and the cisterna magna, although the fastigium and vermis are normal (only an upward rotation of the vermis is observed). The tentorium is in a normal position. Many authors consider this anomaly as delayed closure of the fourth ventricle, which can be simply a normal anatomical variant.

### Mega-cisterna magna

Mega-cisterna magna is defined as enlargement of the cisterna magna (to a diameter ≥ 10 mm) when the other structures of the posterior fossa are normal. On axial ultrasound, no communication will be seen between the fourth ventricle and the cisterna magna and the transverse measurement of the cisterna magna will be ≥ 10 mm, whereas sagittal ultrasound will show that the vermis, fastigium, and tentorium are normal, an enlarged cisterna magna being the only abnormality observed.

### Posterior fossa malformations without cisterna magna enlargement

The posterior fossa malformations in which the cisterna magna is of a normal size include cerebellar hypoplasia, rhombencephalosynapsis, and vermian agenesis, as well as the others listed below. The differential diagnosis will depend on the identification of the characteristics of each malformation previously described through the multiplanar analysis of these structures.

***Arachnoid cyst* -** An arachnoid cyst is defined as a cerebrospinal fluid collection that has no communication with the cisterna magna. Although ultrasound shows an increase in the dimensions of the cisterna magna region, extrinsic compression of the cerebellum is observed (the cerebellar hemispheres can appear asymmetrical on images obtained in the axial plane).

**Figure 9:**
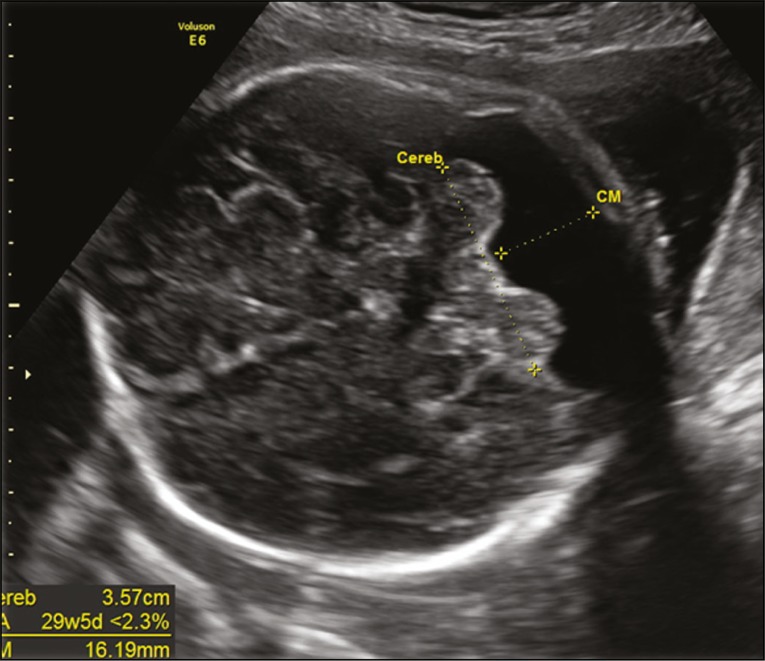
Ultrasound of a fetus with cerebellar hypoplasia, showing a transcerebellar diameter below the 10th percentile for gestational age. The cisterna magna may falsely appear to be increased because the cerebellum is small.

***Cerebellar hypoplasia* -** Cerebellar hypoplasia (typically diagnosed in the third trimester of gestation) is characterized by a smaller-than-normal cerebellum. Ultrasound shows a transcerebellar diameter below 10th percentile for gestational age. The cisterna magna may appear to be falsely increased by the fact that the cerebellum is small (Figure 9).

***Pontocerebellar hypoplasia* -** Pontocerebellar hypoplasia is defined as a smaller-than-normal cerebellum together with a flat (thin) pons. The diagnosis can be difficult to make by ultrasound and is suspected only when the transverse diameter of the cerebellum is small for gestational age and the pons is flat.

**Figure 10:**
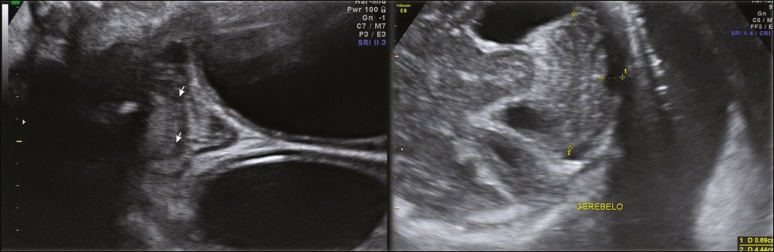
Coronal ultrasound of a fetus with rhombencephalosynapsis, showing that the foliations of the cerebellar hemispheres are continuous in the middle portion of the cerebellum, and that there is no observable vermis (arrowheads). Axial ultrasound can show a cerebellum with a transverse diameter that is normal or below the 10th percentile for gestational age and with a triangular shape.

***Rhombencephalosynapsis* -** Rhombencephalosynapsis is described as fusion of the cerebellar hemispheres, together with different degrees of vermian hypoplasia/agenesis. Axial ultrasound usually shows a cerebellum with a transverse diameter that is normal or below the 10th percentile for gestational age and with a triangular shape. In the coronal plane, the foliations of the cerebellar hemispheres are continuous in the middle portion of the cerebellum and no vermis is observed (Figure 10).

***Vermian agenesis* -** Vermian agenesis is defined as complete absence of the cerebellar vermis. Ultrasound diagnosis in the prenatal period is often difficult. One example is Joubert syndrome, an autosomal recessive disorder characterized by vermian agenesis, mental retardation, ataxia, and abnormal behavior.

***Unilateral cerebellar lesions* -** Unilateral cerebellar lesions is characterized by total or partial destruction of the cerebellum. Such lesions are due to a prenatal insult (infarction, infection, or hemorrhage).

## CONCLUSION

Despite all of the standardization described herein, the diagnosis of posterior fossa malformations can still be difficult in some cases. We have to bear this in mind and emphasize it in the couple counseling sessions. In such cases, serial ultrasound evaluations might be necessary during pregnancy, as might the use of complementary diagnostic methods such as magnetic resonance imaging (MRI). Although the real advantage of MRI over ultrasound is debated^(10,11)^, it is known that MRI allows better evaluation of the position of the torcula, which is an important marker for the differential diagnosis of cystic malformations of the posterior fossa^(3)^.

Although fetal malformations of the posterior fossa encompass a heterogeneous group of abnormalities, they can have similar characteristics on ultrasound. The method of choice in the screening for and diagnosis of these anomalies in the prenatal period is multiplanar ultrasound (including neurosonographic evaluation with high-frequency probes, a transvaginal approach being used for fetuses in vertex presentation). The correct diagnosis of these malformations is very important for the counseling and follow-up of such pregnancies.
